# The COVID-19 Pandemic Enhanced Virology Research in Greece

**DOI:** 10.3390/v15010069

**Published:** 2022-12-25

**Authors:** Apostolos Beloukas, Serafeim C. Chaintoutis, Ioannis Karakasiliotis

**Affiliations:** 1 Department of Biomedical Sciences, School of Health Sciences, University of West Attica, 12243 Athens, Greece; 2 National AIDS Reference Centre of Southern Greece, School of Public Health, University of West Attica, 11521 Athens, Greece; 3 Diagnostic Laboratory, School of Veterinary Medicine, Faculty of Health Sciences, Aristotle University of Thessaloniki, 54627 Thessaloniki, Greece; 4 Medical School, Democritus University of Thrace, University Campus Dragana, 68100 Alexandroupolis, Greece

The emergence of the novel severe acute respiratory syndrome coronavirus 2 (SARS-CoV-2) presented an unprecedented public health threat, being the cause of one of the most devastating pandemics in history. The coronavirus disease (COVID-19) pandemic has emphasized global connectivity, vulnerability, and inequities. The global response to the pandemic has been suboptimal and foregrounded protectionism versus global cooperation, and politics versus public health. Nevertheless, the collaborative research response has played a critical and frontline role in understanding the novel SARS-CoV-2 and combatting the COVID-19 pandemic. Humanity must learn its lesson by how the world responded to this pandemic, as it will most certainly not be the last one to challenge the world.

The urgent need to study and tackle the COVID-19 pandemic also greatly boosted virology research in Greece, with groups from the field and from other disciplines joined their efforts to focus on analyzing molecular, clinical, and epidemiological aspects of SARS-CoV-2 [1]. Such efforts further enhanced virology research in Greece, increasing the use of state-of-the-art technologies as the legacy of COVID-19 research.

In this Special Issue, contributing research groups analyzed the kinetics and avidity of SARS-CoV-2 antibody responses in hospitalized and nonhospitalized COVID-19 patients [2], as well as the role of SARS-CoV-2 antigenemia/viremia in masking the ability of immunodiagnostic assays to detect humoral responses [3]. The structural analysis of the receptor-binding domain (RBD) of the spike (S) protein and angiotensin-converting enzyme (ACE2) presented a potential effectiveness of bisartans as SARS-CoV-2 inhibitors [4]. The analysis of amino acid substitution accumulation revealed common and distinctive amino acid substitution patterns in several SARS-CoV-2 variants of concern [5], while the contribution of mutations and recombination were discussed in a thorough review on the evolutionary plasticity of coronaviruses [6]. The management of the pandemic presented a significant challenge for all countries worldwide. In a study evaluating publicly available data concerning the first three waves of the pandemic, researchers identified country-level risk factors responsible for the spread of SARS-CoV-2 [7].

The work on SARS-CoV-2 did not diminish the contemporary study of other clinically important viruses, such as human immunodeficiency virus (HIV), hepatitis C virus (HCV), and human papillomavirus (HPV), which remain a global epidemiological burden. In the present Special Issue, researchers analyzed the global burden of *Cyclospora cayetanensis* infection in associated risk factors in people living with HIV and/or AIDS [8]. A study on body mass index changes after retroviral treatment revealed differences according to the class of the core drug used [9]. The HCV molecular pathology became another important focus of this Special Issue, revealing the genotype-specific role of HCV core protein in the susceptibility of hepatocytes to tumor necrosis factor (TNF) [10] and the differential biological role of two isoforms of HCV Core+1 protein [11]. Work on hepatocyte biochemistry revealed that HCV and dengue virus, another hepatotropic virus, replication is associated with catecholamine biosynthesis [12,13]. The Special Issue concluded with the growing importance of HPV variants, such as the T350G variant, in the understudying of the pathobiology of oropharyngeal cancer [14], as well as the assessment of the prevalence rates of common viruses, such as papilloma, herpes, and pox viruses, on the skin of beach volley athletes [15].

To conclude, this Special Issue represents a collection of articles studying and discussing different aspects of state-of-the-art virus research in Greece, from the molecular biology of viruses and pathogenesis of associated infections to antiviral agents, the effectiveness of antiviral therapy, and, finally, response to vaccination. We hope that the contents of this Special Issue help readers and researchers take forward steps in cutting-edge virology research, and further enhance interdisciplinary collaborations.

## Data Availability

Not applicable.
